# Respiratory Infections during SARS Outbreak, Hong Kong, 2003

**DOI:** 10.3201/eid1111.050729

**Published:** 2005-11

**Authors:** Janice Y.C. Lo, Thomas H.F. Tsang, Yiu-Hong Leung, Eugene Y.H. Yeung, Thomson Wu, Wilina W.L. Lim

**Affiliations:** * Department of Health, Hong Kong Special Administrative Region, People's Republic of China

**Keywords:** SARS, effect, respiratory, infection, community, hygiene, measures, control, influenza, pandemic, dispatch

## Abstract

The effect of community hygienic measures during the outbreak of severe acute respiratory syndrome in Hong Kong was studied by comparing the proportion of positive specimens of various respiratory viruses in 2003 with those from 1998 to 2002. Community hygienic measures significantly reduced the incidence of various respiratory viral infections.

Severe acute respiratory syndrome (SARS) is an infection caused by a novel coronavirus that is transmitted primarily through direct mucous membrane contact with infectious respiratory droplets and through exposure to fomites. In 2003, Hong Kong reported SARS cases from March 11 to June 2. During the height of the outbreak, schools were suspended, social activities were curtailed with the closure of various public places, and the community was engaged in a sustained and intense hygiene campaign (*1**–**3*). Population education on personal hygienic measures was spearheaded by the government with concerted efforts from various organizations and the community. Surveys conducted in April and May 2003 showed that most of the population wore a face mask (76%), washed their hands after contact with potentially contaminated objects (65%), used soap when washing hands (75%), covered their mouths when sneezing or coughing (78%), and used diluted bleach for household cleaning (>50%) (*4**,**5*). Another survey on health-seeking behavioral traits conducted in June 2003 showed that >70% of respondents practiced some of these hygienic measures more frequently during the SARS outbreak than during the pre-SARS period (*6*).

## The Study

We postulated that these populationwide anti-SARS measures would have effects on other infections spread by the respiratory route. In this study, we examined whether such measures also affected the incidence of some common acute viral respiratory infections.

The study period was January 1, 1998, to December 31, 2003. Data were obtained from the Government Virus Unit (GVU), a public health and diagnostic virology laboratory serving public and private hospitals and outpatient clinics in Hong Kong. At GVU, all respiratory specimens are routinely cultured using 4 continuous cell lines, Rhesus monkey kidney, Madin-Darby canine kidney, rhabdomyosarcoma, and human laryngeal epithelium, which could support the growth of various viruses including influenza, parainfluenza, respiratory syncytial virus (RSV), and adenovirus. On detection of specific cytopathic effects, the viruses are identified with standard protocols (*7*).

For each month of the study period, we obtained the number of respiratory virus isolates as a proportion of the total number of respiratory specimens processed by GVU. We computed the percentage change in the proportion of positive specimens (PPS) for each virus between each month of 2003 and the mean PPS in the same month of the preceding 5 years (1998–2002), which served as the reference period. For comparison purposes, we obtained the monthly number of positive tests for immunoglobulin M (IgM) antibody against hepatitis B core antigen (anti-HBc) and the corresponding total number of tests performed in the study period. The percentage change in PPS was calculated as above. Although a positive IgM anti-HBc test result indicates acute hepatitis B infection or an exacerbation of chronic hepatitis B infection, as we were testing the same catchment of population throughout 1998 to 2003, the proportion of exacerbations of chronic hepatitis B infection is assumed to have remained unchanged during the study period.

**Figure 1:**
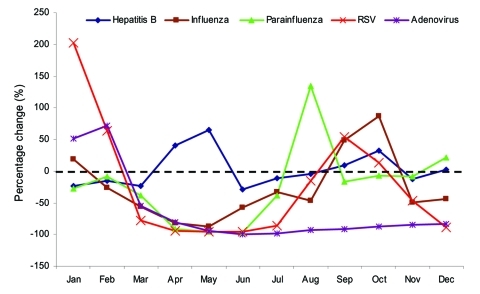
Change in proportion of positive specimens in 2003 for various viruses with reference to period 1998–2002. RSV, respiratory syncytial virus.

The Table shows the change in PPS in 2003 for the various viruses in comparison with the reference period. In 2003, the monthly number of respiratory specimens ranged from 665 to 5,432 (mean 1,399), in comparison with a range throughout the years 1998–2002 of 757 to 3,162 (mean 1,334.5). A surge in the number of specimens was noted during March and April 2003 (5,432 and 3,758, respectively). During March to July 2003, marked reductions in PPS occurred compared with the reference period for influenza virus, parainfluenza virus, RSV, and adenovirus, particularly in the months of April, May, and June. This reduction corresponded to the period when anti-SARS measures in the community were most rigorous. In contrast, similar changes in PPS were not observed for hepatitis B, which is caused by a bloodborne virus with a different mode of transmission than that of the 4 respiratory viruses (Figure 1). Since August 2003, instead of reductions in PPS, a rebound in isolation rates was observed for the 4 viruses.

**Table:** Change in proportion of positive specimens (PPS) in 2003 for various viruses with reference to the period 1998–2002*

Virus		Jan	Feb	Mar	Apr	May	Jun	Jul	Aug	Sep	Oct	Nov	Dec
Influenza													
	No. isolates	327	419	786	105	22	58	96	54	121	50	10	35
	No. tests performed	1,166	1,530	5,432	3,758	1,495	974	776	665	1,283	1,193	1,008	1,407
	PPS (03)	0.28	0.27	0.14	0.03	0.01	0.06	0.12	0.08	0.09	0.04	0.01	0.02
	Average PPS (98–02)	0.235	0.368	0.329	0.154	0.127	0.143	0.186	0.153	0.063	0.022	0.019	0.044
	Range of PPS (98–02)	0.067–0.321	0.143–0.519	0.121–0.517	0.065–0.203	0.043–0.194	0.072–0.216	0.145–0.232	0.123–0.173	0.040–0.105	0.009–0.044	0.008–0.030	0.022–0.110
	% change†	+19	–26	–56	–82	–88	–58	–33	–47	+49	+87	–49	–44
Parainfluenza													
	No. isolates	29	35	75	12	2	1	11	26	29	68	75	112
	No. tests performed	1,166	1,530	5,432	3,758	1,495	974	776	665	1,283	1,193	1,008	1,407
	PPS (03)	0.02	0.02	0.01	0.00	0.00	0.00	0.01	0.04	0.02	0.06	0.07	0.08
	Average PPS (98–02)	0.035	0.025	0.022	0.036	0.037	0.034	0.023	0.017	0.027	0.061	0.081	0.065
	Range of PPS (98–02)	0.014–0.106	0.014–0.053	0.008–0.046	0.021–0.046	0.024–0.054	0.026–0.046	0.017–0.034	0.010–0.028	0.014–0.043	0.025–0.102	0.031–0.149	0.035–0.156
	% change†	–28	–8	–38	–91	–96	–97	–38	+135	–16	–7	–9	+22
RSV													
	No. isolates	49	53	66	24	6	3	8	53	181	48	12	5
	No. tests performed	1,166	1,530	5,432	3,758	1,495	974	776	665	1,283	1,193	1,008	1,407
	PPS (03)	0.04	0.03	0.01	0.01	0.00	0.00	0.01	0.08	0.14	0.04	0.01	0.00
	Average PPS (98–02)	0.014	0.021	0.055	0.104	0.081	0.066	0.072	0.094	0.092	0.036	0.022	0.032
	Range of PPS (98–02)	0.003–0.054	0.010–0.065	0.037–0.090	0.055–0.155	0.049–0.112	0.042–0.098	0.038–0.113	0.046–0.126	0.057–0.118	0.018–0.051	0.001–0.060	0.001–0.072
	% change†	+203	+64	–78	–94	–95	–95	–86	–15	+54	+13	–47	–89
Adenovirus													
	No. isolates	61	78	84	41	5	0	1	2	4	7	8	17
	No. tests performed	1,166	1,530	5,432	3758	1,495	974	776	665	1,283	1,193	1,008	1,407
	PPS (03)	0.05	0.05	0.02	0.01	0.00	0.00	0.00	0.00	0.00	0.01	0.01	0.01
	Average PPS (98–02)	0.034	0.030	0.035	0.056	0.055	0.058	0.068	0.044	0.038	0.049	0.052	0.074
	Range of PPS (98–02)	0.012–0.058	0.019–0.052	0.017–0.071	0.012–0.121	0.022–0.110	0.028–0.105	0.032–0.091	0.029–0.058	0.026–0.054	0.045–0.055	0.042–0.062	0.058–0.093
	% change†	+52	+72	–55	–81	–94	–100	–98	–93	–92	–88	–85	–84
Hepatitis B													
	No. of positive test results	16	11	13	18	20	13	19	20	22	21	15	20
	No. of tests performed	143	98	135	99	97	118	139	135	120	111	102	131
	PPS (03)	0.112	0.112	0.096	0.182	0.206	0.110	0.137	0.148	0.183	0.189	0.147	0.153
	Average PPS (98–02)	0.147	0.132	0.127	0.129	0.125	0.156	0.154	0.156	0.168	0.144	0.167	0.148
	Range of PPS (98–02)	0.083–0.228	0.079–0.190	0.093–0.153	0.070–0.168	0.085–0.162	0.096–0.188	0.104–0.186	0.068–0.228	0.086–0.250	0.081–0.233	0.099–0.249	0.082–0.197
	% change†	–24	–15	–24	+41	+65	–29	–11	–5	+9	+32	–12	+3

*RSV, respiratory syncytial virus. †Percentage change of PPS in 2003 with respect to the average PPS from 1998 to 2002.

**Figure 2:**
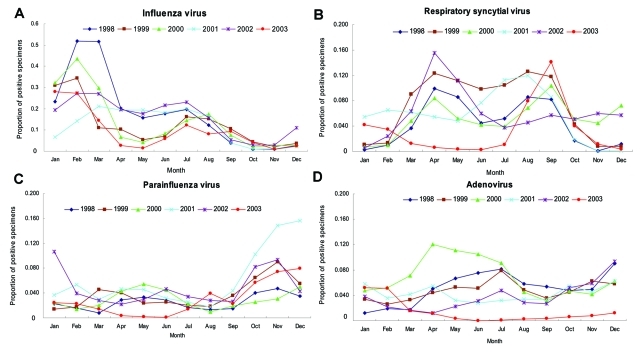
Proportion of positive specimens by month, 1998–2003, for A) influenza virus, B) respiratory syncytial virus, C) parainfluenza virus, and D) adenovirus.

The 2003 SARS outbreak overlapped with the traditional seasonal peak from March to September for RSV in Hong Kong (*8*). In 2003, the RSV peak season shifted to August–October. The accumulation of susceptible infants offset the infection control measures instituted against respiratory infections as well as the normal seasonality; as a result, RSV activity increased in the late months of 2003. Figure 2 illustrates the usual seasonal variation of the 4 respiratory viruses and their pattern from 1998 to 2003.

Discerning whether the observed effects in our study were real or apparent is important. The surge in specimens during March and April 2003 suggested that physicians were more inclined to order tests for patients with respiratory symptoms at the height of the SARS outbreak. This fact could conceivably dilute PPS for respiratory viruses. However, since May 2003, the number of tests has returned to normal levels; however, PPS remained significantly decreased during May to July 2003. Thus, PPS reductions cannot be explained by a dilution effect caused by an increased number of specimens processed. Furthermore, after we controlled for the patients' age group differences, PPS for influenza virus remained depressed when compared to PPS in the reference period (data not shown). The same pattern was true for adenovirus.

Population coverage for influenza vaccination in Hong Kong has been <15% throughout the study period (A. Chan, pers. comm.), so vaccination was unlikely to have resulted in reduced influenza circulation in the community. The concomitant significant reduction in PPS for all 4 respiratory viruses in the same period argues against 2003's being a milder year for influenza. Temporally, the moderation of PPS reductions since August 2003 (the last SARS case was reported on June 2) supported the hypothesis that the effects of populationwide anti-SARS measures on the incidence of respiratory viruses were real.

With the recent outbreaks of highly pathogenic avian influenza among poultry in Asian countries, and the associated human infections, pandemic planning for influenza has been undertaken with renewed efforts on a worldwide basis (*9*). In pandemic preparedness planning, control measures have traditionally focused on the use of antiviral chemotherapy and the expedient development of an effective vaccine. However, such strategies may not be feasible, especially in countries with limited resources. An effective vaccine would probably become available only during the latter phase of the pandemic. Information concerning the effects of increased social distance and communitywide hygiene measures on the incidence of common viral respiratory infections at a population level has been lacking. The SARS outbreak offers a unique opportunity to study the association. Although our study was observational and thus could not establish a causal relationship, it suggests a possible association between population-based hygienic measures and the reduced incidence of influenza and other acute viral respiratory infections. However, the relative contribution of each of these measures could not be estimated in our study. The effective implementation of such measures requires determined and sustained educational efforts from health authorities with collaboration of the public. We thus propose that stockpiling personal protective equipment and having public education campaigns on infection control practices should form an integral component in pandemic planning for the emergence of novel influenza virus strains in humans.
